# MicroRNA in Cervical Cancer: OncomiRs and Tumor Suppressor miRs in Diagnosis and Treatment

**DOI:** 10.1155/2014/178075

**Published:** 2014-01-02

**Authors:** Kouji Banno, Miho Iida, Megumi Yanokura, Iori Kisu, Takashi Iwata, Eiichiro Tominaga, Kyoko Tanaka, Daisuke Aoki

**Affiliations:** Department of Obstetrics and Gynecology, School of Medicine, Keio University, Tokyo 160-8582, Japan

## Abstract

Cervical cancer is a female-specific disease with a high incidence and mortality. MicroRNAs (miRNAs) are implicated in posttranscriptional regulation of gene expression and in the pathogenic mechanisms of cancer, suggesting their importance in diagnosis and treatment. miRNAs may have roles in the pathogenesis of cervical cancer based on the increases or decreases in several specific miRNAs found in patients with this disease. The miRNAs implicated in cervical cancer are miR-21, miR-126, and miR-143, and clinical application of these miRNAs for diagnosis and treatment is under investigation. Methods for diagnosis of cervical cancer include analysis of changes in the levels of specific miRNAs in serum and determination of aberrant hypermethylation of miRNAs. Supplementation of miR-143 or inhibition of miR-21 activity *in vivo* may be therapeutic strategy for cervical cancer. Previous approaches to development of siRNA as a drug have provided information for establishment of therapy based on these approaches, and an anti-miR-21 inhibitor has been developed. miRNAs also have effects on drug resistance and may be useful in combination therapy with other drugs.

## 1. Introduction

Cervical cancer is a primary cancer of the uterine cervix in females and has a high incidence and mortality. The mortality of cervical cancer is decreasing in countries with advanced healthcare systems, but the incidence is still high in developing countries and the disease is the second most common female cancer worldwide, with an estimated global incidence of 470,000 new cases and approximately 233,000 deaths per year [1]. In Japan, the incidence and mortality of cervical cancer are increasing in women aged 30 to 40 years old [2]. Prevention, diagnosis, and treatment of female-specific diseases are increasingly important issues due to lifestyle changes of women, including workforce participation and increased life expectancy.

## 2. Mechanism of MicroRNA in Carcinogenesis

MicroRNAs (miRNAs) are involved in cancer pathogenesis by posttranscriptional regulation of gene expression. Many studies have examined the use of miRNAs as cancer diagnostic marker and as anticancer therapy. miRNAs are small RNA molecules of 20–23 nucleotides that do not encode a protein. miRNAs involved in carcinogenesis are classified into oncogenic miRNAs (oncomiRs) and tumor suppressor miRNAs (tumor suppressor miRs) [3]. Processing of miRNA generally starts from transcription by RNA polymerase II to a primary miRNA (pri-miRNA). This primary transcript is cleaved into pre-miRNAs by the nuclear RNase III Drosha, and then the pre-miRNA is transformed to a mature miRNA by Dicer, another RNase III. miRNAs of 20–23 nucleotides bind the complementary sequences in the 3′ untranslated region (UTR) of a target mRNA, which represses translation and enhances degradation of the target and results in negative regulation of gene expression. The mechanism of miRNA repression of translation of target genes results in blocking the effect of eukaryotic initiation factors, which bind to mRNAs by recognition of the 5′ tail cap structure of the mRNA. Poly A-binding protein (PABP) binds to the 3′ poly A tail of the mRNA to attract eIF4G to mRNAs, and thus translation is initiated by synergistic action of the mRNA cap structure and poly A tail. The RNA-induced silencing complex (RISC) containing a miRNA binds the 3′-UTR in mRNAs and induces formation of the CCR4-CAF1-NOT complex, a poly A tail-truncating enzyme, resulting in truncation of the downstream poly A tail, reduced binding of translation initiation factors, and repression of translation [4].

## 3. Human Papillomavirus-Induced Carcinogenesis and *p53*

Cervical cancer mostly occurs due to long-term infection with human papillomavirus (HPV). HPV infection associated with sexual intercourse is common. HPV invades basal cells of the cervix through mucosa and skin damaged by intercourse and is maintained as an episome. Once viral DNA is incorporated into host DNA, oncogenic transformation is induced [5]. This incorporation has a characteristic pattern, in which the *E6* and *E7 *genes of the viral DNA are maintained, but the *E2* gene is deleted [6]. *E6* and *E7* inactivate tumor suppressor genes *p53 *and* pRB*, respectively, and persistent HPV infection induces cell neoplastic transformation. High-risk HPV can induce immortalization and neoplastic transformation because *E6* and *E7* are incorporated into host DNA and are persistently overexpressed. E6 protein binds to E6-associated protein (E6-AP), a ubiquitin protein ligase (E3), and the E6/E6-AP complex binds to a target protein and induces degradation through the ubiquitin-proteasome system. The most common target of the E6/E6-AP complex is the tumor suppressor gene *p53*. E6 degrades p53, inhibits p53 binding with DNA, and binds to *p53 *enhancers, thereby suppressing p53 function. Thus, E6 blocks the *p53* pathway in multiple ways [7]. The E6/E6-AP complex also activates telomerase and contributes to cell immortalization [8] and is implicated in malignant progression and cell tumorigenicity by enhancing degradation of PDZ (PDS-95, disc-large, ZO-1) proteins [9]. E6 also binds apoptosis inducers and inhibits this function [7], while the E7 protein contributes to carcinogenesis by degrading Rb family proteins necessary for cell cycle progression [10].

## 4. *p53*-Related miRNA

Recent studies have shown that miRNA-induced abnormal regulatory mechanisms are implicated in the pathogenesis of many diseases, including malignant tumors. miRNAs are classified into oncogenic and tumor suppressor miRNAs [11] and the miRNA expression level frequently decreases in human cancer tissues [12]. Thus, many miRNAs may have the potential for tumor suppression, including miRNAs associated with the *p53* gene.

Georges et al. and Braun et al. separately found that miR-192, miR-194, and miR-215 are induced by DNA damage in a *p53*-dependent manner [13, 14], while Yan et al. identified the miR-17-92 cluster as a miRNA repressed by *p53* under hypoxic conditions [15]. Screening for miRNAs regulating the *p53* pathway showed that the miR-29 family (miR-29a, miR-29b, and miR-29c) activates the *p53* pathway [16]. In addition, miR-122 positively regulates the *p53* pathway via cyclin G1 [17], miR-125b directly inhibits *p53 *[18], and miR-21 negatively regulates the *p53* pathway by targeting heterogeneous nuclear ribonucleoprotein K (HNPRK), a positive regulator of the *p53* pathway, and the *p53* homolog, *p63* [19]. miRNA can also affect the downstream *p53* pathway, based on the negative regulation of *p53*-induced CDK inhibition by miR-372 and miR-373 via LATS2 [20].

## 5. Changes in miRNA Expression in Cervical Cancer

Large-scale miRNA microarray analysis of clinical specimens is effective for the evaluation of miRNA expression changes. This analysis can be used to compare normal and cancer tissues, primary lesions and metastatic/recurrent lesions, and miRNA expression profiles before and after anticancer treatment. Large-scale microarray analyses have shown specific miRNA expression in particular cancer types [12]. Further examinations of microarray results using quantitative RT-PCR in clinical specimens have suggested relationships of miRNAs with cancer characteristics. By comparing miRNA expression in normal and cancer tissues, many miRNAs with cancer-specific upregulation (oncomiRs) or downregulation (tumor suppressor miRs) have been found.

Many oncomiRs have been discovered in cervical cancer. Among them, miR-21 is overexpressed in many kinds of cancer and is a negative regulator of expression of the tumor suppressor gene* programmed cell death 4 *(*PDCD4*) (Table 1). Expression of *PDCD4* is implicated in apoptosis and also blocks translation and inhibits tumor growth, and thus miR-21 binding to the *PDCD4* 3′ UTR enhances cell growth [21]. miR-21 also targets and regulates *CCL20*, which is involved in tumor differentiation and nodular metastasis [22]. miR-10a enhances tumor growth, metastasis, and invasion by suppressing *CHL1* expression [23], while miR-19a and miR-19b are overexpressed in cervical cancer cells and implicated in cytopoiesis of malignant-type HeLa and C33A cells. miR-19a and miR-19b enhance cell growth and invasion, whereas miR-19a and miR-19b null cells are nonmalignant. *Cullin 5 (CUL5)* is targeted by both miR-19a and miR-19b, but expression of *CUL5* excluding the 3′ UTR decreases cell growth and invasion induced by miR-19a and miR-19b [24]. miR-20 enhances expression of the oncogene *tyrosine kinase*, *nonreceptor 2 (TNKS2)*. Once *TNKS2* is suppressed, metastasis and invasion of cervical cancer cells are suppressed. However, miR-20 positively regulates *TNKS2*, resulting in enhanced metastasis and invasion [25]. miR-133b regulates *mammalian sterile 20-like kinase (MST2)*, *cell division control protein 42 homolog (CDC42)*, and *ras homolog gene family member A (RHOA)* and consequently activates the mitogen-activated protein kinase 1 (MAPK1) and v-akt murine thymoma viral oncogene homolog 1 (AKT1) signaling pathways and facilitates malignant transformation. Carcinogenesis and metastasis in the lung were produced by injecting cervical cancer cells with overexpression of miR-133b [26]. The latency period of cervical neoplasm is shortened as miR-133b expression is increased, which makes miR-133b a marker for early cervical cancer.

Many studies have also examined tumor suppressor miRNAs. For example, miR-138 is involved in regulation of telomerase, an enzyme that is strongly associated with cell immortalization and carcinogenesis through extension of telomeres in chromosomal ends. Telomerase activity depends on expression of *human telomerase reverse transcriptase (hTERT)*, and miR-138 suppresses the *hTERT* mRNA level and reduces telomerase activity. miR-138 expression in cervical cancer cells is significantly lower than that in normal tissues, which causes telomerase activation and carcinogenesis [27] (Table 1). In HeLa and C33-A cervical cancer cells, overexpressed miR-7 inhibits cell growth and enhances apoptosis, whereas suppression of miR-7 expression has opposite effects. miR-7 targets and regulates *X-linked inhibitor of apoptosis protein (XIAP)* [28]. Therefore, miR-7 is a tumor suppressor miRNA. miR-17-5p targets *tumor protein p53-induced nuclear protein1 (TP53INP1)* and inhibits cell growth, leading to apoptosis. *TP53INP1* plays an important role in stress reactions in cells and miR-17-5p-induced cell growth inhibition is interfered if *TP53INP1* is ectopically expressed. A similar relationship between *TP53INP1* and miR-17-5p has been found in cervical cancer tissues. Therefore, miR-17-5p may also be a tumor suppressor miRNA [29]. Cui et al. analyzed cell growth and apoptosis in cervical cancer cells by MTT assay and flow cytometry and showed that overexpression of miR-125b inhibited cell growth, induced apoptosis, and decreased tumorigenicity by suppression of the *phosphoinositide 3-kinase catalytic subunit delta (PIK3CD)* through targeting of the PI3 K/Akt/mTOR signaling pathway [30].

## 6. Diagnosis of Cervical Cancer Using miRNAs in Serum

RNAs degrade quickly when transferred into blood due to the presence of RNases. Therefore, miRNAs were initially thought not to exist in serum and miRNAs in cells have commonly been used for miRNA expression analyses. However, secreted miRNAs embedded in exosomes and granular vesicules circulating in blood were discovered in 2007 [31]. Such miRNAs have subsequently been found to have roles in transfection through intercellular communication [32] and in signal transduction through transfer via the placenta and breast milk [33].

miRNAs secreted extracellularly are also of interest in cancer diagnosis and treatment because secreted miRNA profiles of patients with cancer differ significantly from those of normal volunteers. Reagents for extraction of miRNAs from plasma and serum are available and miRNA expression can be examined by microarray analysis and quantitative RT-PCR. miRNAs with specific expression changes in cancer have the potential to be diagnostic and prognostic biomarkers based on plasma and serum tests [34]. miR-126 and miR-21 are found in serum and are associated with cervical cancer [35–37]. Other new candidate miRNA markers were found in an analysis of serum collected from patients with cervical cancer. Overexpression of miR-21, miR-27a, miR-34, miR-34a, miR-146a, miR-155, miR-196a, miR-203, and miR-221 was detected, with miR-21, miR-27a, miR-34, miR-34a, and miR-196a being particularly highly expressed in squamous cell carcinoma of the cervix [38]. These results indicate that miRNA levels in serum can be used for diagnosis of cervical cancer and that some miRNAs may be useful for prediction of tissue type and for early diagnosis of cervical cancer.

## 7. Diagnosis of Lymph Node Metastasis Using miRNAs in Serum

Several miRNAs in serum have been identified as candidate markers for lymph node metastasis in cervical cancer. Zhao et al. analyzed expression of miR-20a and miR-203 in serum collected before surgery and treatment in 80 patients with FIGO stages I to IIA cervical cancer. The miR-20a level in serum of patients with cervical cancer was markedly higher than that in healthy volunteers and was overexpressed in patients with lymph node metastasis. The expression level of miR-203 in patients with cervical cancer showed a tendency to be higher in comparison with that in healthy volunteers; however, lymph node metastasis was found only when miR-203 expression was suppressed. Based on groups of patients with and without lymph node metastasis, miR-20a in serum had an area under the curve (AUC) of 0.734 ± 0.058, sensitivity of 75%, specificity of 72.5%, and a cut-off value of 3.0 as a marker for metastasis. Using the same groups of patients, miR-203 had an AUC of 0.658 ± 0.061 and was a less effective marker than miR-20a, with sensitivity of 65%, specificity of 62.5%, and a cut-off value of 0.13 [39]. Zhao et al. also showed that overexpression of miR-20a was related to histological grade and tumor size [40].

Chen et al. conducted a study of miRNA markers of lymph node metastasis in early cervical cancer in 40 patients with cervical cancer with lymph node metastasis, 40 patients with cervical cancer without lymph node metastasis, and 20 healthy volunteers. miRNA expression levels were measured by qPCR and a ROC curve was determined. The candidate markers for lymph node metastasis were miR-1246, miR-20a, miR-2392, miR-3147, miR-3162-5p, and miR-4484. miRNA in serum had an AUC of 0.932, sensitivity of 85.6%, and specificity of 85.0% for prediction of lymph node metastasis. For miRNA in tissue, the AUC was 0.992, sensitivity was 96.7%, and specificity was 95.0%. A ROC curve for SCC antigen in serum as a predictor for lymph node metastasis had an AUC of 0.713, sensitivity of 61.2%, and specificity of 70.0%. Therefore, miRNA in serum was superior to SCC antigen in serum for the prediction of lymph node metastasis [41].

Huang et al. conducted a study of miRNA in patients with small cell carcinoma of the cervix (SCCC). Expression levels of 7 miRNAs, let-7c, miR-10b, miR-100, miR-125b, miR-143, miR-145, and miR-199a-5p, were markedly suppressed in patients with advanced SCCC (FIGO2008 I stage B2-IV) in comparison with those in patients with early SCCC (FIGO2008 I stage B1). In patients with lymph node metastasis, expression of let-7c, miR-100, miR-125b, miR-143, miR-145, and miR-199a-5p was particularly suppressed. Kaplan-Meier survival curves showed that patients with hypoexpression of miR-100 and miR-125b had a tendency for a poor prognosis [42]. These results suggest that screening for miRNAs can be effective for identification of lymph node metastasis in early cervical cancer and for prediction of prognosis in advanced stage disease.

## 8. Diagnosis of Cervical Cancer Using Aberrant Hypermethylation of miRNAs

Some miRNAs are downregulated by epigenetic modifications involving DNA hypermethylation. A demethylating agent such as 5-aza-2′-deoxycytidine (5-aza-dC) can reverse DNA hypermethylation and cause expression changes in miRNA microarray analysis. Of miRNAs downregulated in cervical cancer, the change in miR-124 involves aberrant hypermethylation [43]. Aberrant hypermethylation of miRNAs can be analyzed by methylation specific PCR (MSP) and combined bisulfite restriction analysis (COBRA). Therefore, aberrant hypermethylation of miR-124 shown by MSP can be used for diagnosis in patients suspected to have cervical cancer. Levels of other miRNAs may also be altered by aberrant hypermethylation in cervical cancer. Yao et al. examined HPV-infected cells in the uterine cervix and found that the genes encoding miR-432, miR-1286, miR-641, miR-1290, miR-1287, and miR-95 were hypermethylated [44]. Hypermethylation of miR-149, miR-203, and miR-375 has also been found in HPV-positive high-grade dysplasia [45], and miR-203 and miR-375 may be markers for precancerous conditions in the uterine cervix. These and other miRNAs show different changes in cervical cancer, and analysis of a combination of miRNAs may be most appropriate for diagnosis, perhaps based on a scoring system for multiple miRNAs.

## 9. Treatment with miRNA Supplementation

Anticancer treatment may be achieved by regulating the expression level of miRNAs. This could include inhibiting the function of miRNAs (oncomiRs) overexpressed in cancer by administration of complementary nucleic acid sequences or by decreasing the actual expression level. In contrast, the function of miRNAs (tumor suppressor miRs) with reduced levels in cancer may be recovered by supplementation of the miRNA itself. Both strategies use synthetic nucleic-acid-based drugs that require delivery systems. Similar delivery of synthetic nucleic acids is also an important issue in development of siRNAs as therapeutic agents, and difficulties in delivery are a reason why *in vitro* effects are often not achieved *in vivo*. A delivery system is required to ensure stability *in vivo* and to efficiently and safely introduce nucleic-acid-based drugs into cells.

Atelocollagen, a protein that forms the terminal region (telopeptide) of collagen type I, is being examined as a potential delivery system for nucleic-acid-based drugs [46]. This protein is extracted from calf dermis, purified, and then digested with protease to reduce antigenicity. Atelocollagen is positively charged under physiological conditions and forms a complex with negatively charged nucleic-acid-based agents. This complex can be stably transferred into tissues and cells. This delivery system may be suitable for clinical development because no abnormal gross or biological findings have been found in intravenous administration to rabbits and monkeys at high dose. An inhibitory effect of miR-143 on metastasis, as described next, has been shown following intravenous administration of a complex with atelocollagen in a mouse model. miR-34 supplementation has been performed using this technique. Delivery to the brain is particularly difficult, but a delivery system may be possible based on RGD peptide, which crosses the blood-brain barrier. Thus, several delivery systems for general purpose, safe, and tissue-specific clinical applications of nucleic-acid-based drugs are under development [47].

A strategy for treatment of cancer with suppressed miRNA expression involves recovery of function by supplementation of the miRNA itself. Extracellular administration of a single-stranded mature miRNA is ineffective, since this molecule cannot be recruited by the RISC complex. Therefore, there is a need for administration of synthetic nucleic acid molecules that can be processed to mature miRNA using the intrinsic cellular mechanism. This procedure has some similarities to gene therapy, but administration of synthetic nucleic acid molecules should be safer than the use of viral vectors. Methodology for development of siRNAs as drugs is useful as a reference because therapy based on miRNA administration uses *in vivo* RNAi functions. Supplementary agents can be classified into two structural types: hairpin single-stranded pre-miRNA, the structure before processed by Dicer; and double-stranded RNA, with a conformation similar to siRNA produced by cleaving the loop of a single-stranded hairpin pre-miRNA. Hairpins need to be delivered as long-strand RNAs of at least 50 bases. However, 2-cyanoethoxymethyl (CEM) technology can be used to synthesize long RNAs, as illustrated by the synthesis of a 110 mer miRNA precursor that had miR-196a activity [48]. Double-stranded RNA is similar to that used for siRNA drugs and is available using established technology.

In contrast to siRNAs, miRNAs may have effects without having full complementarity to a target mRNA; therefore, one miRNA may suppress many targets. This is a disadvantage in a pharmaceutical context since specificity is reduced. However, the multiple targets of evolved miRNAs are not random but are gene groups related to a common function. Therefore, introduction of one miRNA suppresses multiple genes in the same signaling pathway, and one miRNA may have an effect similar to that of simultaneous introduction of multiple siRNAs for these genes [49]. In this context, miR-34 supplementation using double-stranded RNA is under development as a therapeutic approach.

The effect of miR-143 supplementation on inhibition of lung metastasis from osteosarcoma has been shown by Osaki et al. [50]. miR-143 was identified in miRNA microarray analysis of human osteosarcoma cells with and without frequent lung metastasis after transplant of human osteosarcoma cells in mice. miR-143 was the miRNA with the lowest expression in cells with frequent metastasis and inhibited infiltration of human osteosarcoma cell in an *in vitro* assay but had no effect on cell growth. Intravenous administration of miR-143 in an osteosarcoma spontaneous lung metastasis model inhibited lung metastasis *in vivo* but had no effect on primary lesion growth. Matrix metalloprotease 13 (MMP13) was decreased in osteosarcoma cells following supplementation of miR-143; MMP13 expression in osteosarcoma cells of the primary lesion was low in cells with high expression of miR-143; and the expression level of miR-143 in the primary human osteosarcoma lesion was lower in cells with metastasis than in those without metastasis. Therefore, MMP13 upregulation due to miR-143 downregulation promoted lung metastasis, which strongly suggests that supplementation of miR-143 to osteosarcoma cells inhibited the invasive and metastatic potential. Thus, miR-143 is a tumor suppressor miRNA and its supplementation may contribute to treatment of cervical cancer. Liu et al. introduced miR-143 into HeLa cervical cancer cells and showed that cell growth was inhibited and apoptosis was enhanced with increased miR-143 expression. Introduction of anti-miR-143 inhibited the effect of miR-143, but miR-143 expression inhibited tumorigenesis, even if pre-miR-143-treated HeLa cells were injected into female nude mice. *Bcl2* expression increased in tissues with downregulated miR-143 expression in comparison with normal tissue. Overexpressed miR-143 in HeLa cells downregulated *Bcl2*, whereas *Bcl2* expression was upregulated by miR-143 knockdown [51]. Therefore, miR-143 has an association with *Bcl2* and treatment targeting this pathway may be possible.

## 10. Treatment by Inhibition of miRNA Function

One strategy for overexpressed miRNA in cancer is to inhibit the miRNA function using agents with complementary binding to the miRNA. It is generally difficult to use siRNAs for miRNA inhibition due to shortness of the strands. Thus, antisense miRNA oligonucleotides (AMOs) are the most common miRNA inhibitors. Antisense technology developed for drug discovery is a useful basis for this procedure, including use of various modifications for stabilization and delivery. For miR-21, an oncomiR in cervical cancer, anti-miR-21 was developed as a modified 2′-O-methoxyethyl (2′-O-MOE) phosphorothioate antisense agent. After introduction of this AMO into cervical cancer cells, Wang et al. [52] examined miR-21 expression in SiHa cells in real time and evaluated cell growth by MTT assay and cell apoptosis by annexin-V-FITC/PI analysis. The effect of the AMO on tumor suppression was evaluated using a tumor growth curve and immunohistochemistry. miR-21 expression was downregulated and tumor growth was markedly suppressed by the AMO in comparison with a control group. Colonization was also suppressed more markedly in the AMO group, and this group was proapoptotic in flow cytometry. Tumorigenesis was detected in 3 out of 8 cases in the AMO group and 6 out of 8 in the control group, and Ki-67, a cell proliferation marker, decreased in the AMO group and many tumor cells were found to have undergone apoptosis.

miR-21 inhibition may be achieved with other approaches, including miRNA sponges, miRNA erasers, and tough decoys. Studies using expression vectors have the goal of absorbing a specific miRNA and inhibiting its function. Tough decoys may have particularly potent inhibitory activity. Thus, for example, Haraguchi et al. inhibited miR-21 using a tough decoy and recovered expression of *PDCD4*, a target gene of miR-21 [53].

## 11. miRNAs Associated with Therapeutic Resistance for Cervical Cancer

Expression of various miRNAs is up- or downregulated in cervical cancer and these expression levels can increase or decrease sensitivity to chemotherapy and radiotherapy. Phuah et al. showed that the expression patterns of 25 miRNAs, including miR-138, miR-210, and miR-744, altered the sensitivity to 1′S-1′-acetoxychavicol (ACA) and cisplatin [54]. Thus, miRNAs may have an important role in the response to chemotherapy. Lei et al. found that miR-155 negatively regulates the EGF-induced epithelial-mesenchymal transition (EMT), inhibits proliferation, metastasis, and invasion, and increases sensitivity to cisplatin [55]. miR-214 upregulates expression of Bax, caspase-9, caspase-8, and caspase-3, enhances apoptosis and inhibits cell growth, and increases sensitivity to cisplatin by silencing *Bcl2l2* expression [56], and miR-218 induces apoptosis, suppresses tumor growth, and increases sensitivity to cisplatin through the AKT-mTOR signaling pathway in cervical cancer cells [57]. Other miRNAs are implicated in resistance to chemotherapy and radiotherapy. Thus, miR-375 contributes to acquisition of resistance to paclitaxel in cervical cancer cells [58], while Ke et al. showed that miR-181a increases cellular resistance to radiotherapy [59] through negative regulation of proapoptotic protein kinase (PRKCD), which suppresses radiation-induced apoptosis and decreases G2/M stage block.

In cervical cancer, cisplatin may be the drug of choice in cases with miRNA expression which produces tumors that are highly sensitive to cisplatin or if paclitaxel-resistant miRNA is expressed. A detailed understanding of miRNA mechanisms may also permit targeted therapeutic strategies based on miRNA inhibition or supplementation, as described above. If overexpression of a specific miRNA causes resistance to chemotherapy or radiotherapy, this resistance may be reduced by inhibiting the miRNA function. Similarly, if downregulation of a miRNA causes resistance, this may be improved by supplementation of this miRNA. Therefore, new combination therapy of miRNA inhibitors or supplementation with chemotherapy or radiotherapy may be developed. Such treatment approaches using miRNAs with distinct expression patterns may be particularly useful in personalized treatment and molecular targeted therapy for cervical cancer.

## 12. Conclusion

The effects of miRNAs are relatively new concepts, but the possible clinical applications of miRNAs are increasingly recognized. Secreted miRNAs in serum may be useful for cancer diagnosis, including early diagnosis of cervical cancer based on changes in various miRNA serum levels. Examination of aberrant methylation of miRNA genes by MSP and COBRA may also be useful, and the accuracy of diagnosis and stage determination can be improved by combined analyses of various miRNAs. Application to treatment is also becoming realistic based on the knowledge from conventional siRNA drug development. Inhibitors of miR-21, which is overexpressed in cervical cancer, have been developed and inhibition of this miRNA is currently being studied using a new technology, the so-called tough decoy. In contrast, miR-143 is downregulated in cervical cancer, and anticancer effects have been achieved by supplementation of miR-143 after transplant of human osteosarcoma cells in mice. Thus, miRNA supplementation therapy may be possible. Sensitivity to other therapy can also be dependent on expression levels of miRNAs, which suggests that combination therapy using miRNA inhibition or supplementation may be useful. These findings suggest many approaches to miRNA-specific personalized treatment and molecular targeted therapy, and miRNAs are likely to be important in diagnosis and treatment of cervical cancer.

## Figures and Tables

**Table 1:** Expression of microRNAs in cervical cancer cell [1, 4, 38].

Overexpressed miRNAs	Chromosome	Underexpressed miRNAs	Chromosome
miR-15a	13	miR-1	1
miR-15b	3	miR-7	10
miR-16	13	miR-10b	2
miR-17-5p	13	miR-29a	7
miR-19a	4	miR-30b	8
miR-20a	13	miR-100	11
miR-20b	X	miR-124	6
miR-21	17	miR-126	9
miR-27b	9	miR-138	13
miR-93	7	miR-143	5
miR-106a	X	miR-145	5
miR-133b	18	miR-149	2
miR-146	5	miR-194	4
miR-183	8	miR-195	19
miR-185	22	miR-214	1
miR-193b	16	miR-218	1
miR-196a	9	miR-376a	14
miR-199a	19	miR-422a	15
miR-203	14	miR-424	X
miR-210	11	miR-450	X
miR-224	X	miR-451	17
miR-324-5p	17	miR-455	9
miR-372	11	miR-487b	14
miR-373	3	miR-495	14
miR-375	2	miR-574	4
miR-432	14		
miR-503	X		
miR-641	19		
miR-1286	22		
miR-1290	1		
miR-2392	26		
miR-3147	7		
miR-3162-5p	11		
miR-4484	4		
